# The Future of the Deposit Contributor

**Published:** 1914-04-25

**Authors:** 


### NATIONAL INSURANCE ACT DEVELOPMENTS.

### The Future of the Deposit Contributor.

It will be necessary for an Act of Parliament to be passed during this year to make provision for the future of the class of persons, employed within the meaning of the National Insurance Acts, known as Deposit Contributors. This necessity arises from the fact that Section 42 of the principal Act, which brought the Deposit Contributor class into existence, specifically limits the operation of the present arrangements so that they will only apply until January 1, 1915. The imminence of another National Insurance Act is already beginning to attract notice, and there are many features in the problem that confronts the Government which demand the closest attention of all those who are interested in the health and well-being of the community.

Although it is only necessary for the present arrangements regarding Deposit Contributors to be brought under review, it will at once be seen upon closer investigation that many interests are involved in the problem beyond those of the persons directly and immediately concerned. The class in question was created as a temporary expedient and was admittedly an experiment, but like so many temporary expedients it is likely to give rise to problems more difficult of solution than the one that originally confronted its authors.

### The Original Object of the Class.

The main reason for setting up the Deposit Contributor class at the inception of the National Insurance scheme was to relieve the Approved Societies of the burden of accepting as members persons whose health was known to be indifferent, and who would therefore, if admitted, cause a prolonged strain upon the funds and thus do harm to the interests of the general body of members. It was recognised that if the Approved Societies were to be made responsible for the payment of benefits and were each to bear the burden of any deficiency that might arise, it was necessary that they should be given the power to select their members, and not be compelled to accept any applicant irrespective of the state of his health. In point of fact the Approved Societies were given power to admit or reject applications for membership, except that they were not entitled to decline a member solely on account of his age.

It was thus anticipated that the Deposit Contributor class would be mainly composed of those who had been unable to gain admittance to any Approved Society on account of their ill-health, and that they would therefore, as a whole, be distinctly under the average as compared with those who gained admittance as members of Approved Societies. On this ground much sympathy was drawn forth on the woes of the Deposit Contributor, and as the benefits provided for the class are not really insurance, in any proper sense of the word, the Government were freely criticised for giving to those who most needed insurance much less protection than was afforded to those in better health.

As matters have turned out this anticipation has very largely been negatived. There are several reasons for this, and as the reasons bear upon the solution that must ultimately be found it is well that they should be set forth in detail so that they may be fully appreciated.

### Expectations not Realised.

In the first place the almost undue prominence that was given to the disadvantages of becoming a Deposit Contributor, both by the Insurance Commissioners in their official leaflets and by lecturers, had the effect of stimulating the desire for admittance as members of Approved Societies. It must also not be forgotten that just before the Act came into operation the daily press conducted a campaign on similar lines with like results, so that those who in any way anticipated having to draw on the insurance benefits in the near future were induced to become members of Approved Societies. Contrary to the expectation of the framers of the Act, the Approved Societies in their "scramble for members" were only too ready to admit anyone to membership, and it was the exception rather than the rule to demand any evidence, beyond that of a superficial nature, as to the health of those desiring to obtain the full advantages of the insurance. The result was, and still is, that very few of the "bad lives" became, or continue to be, Deposit Contributors.

On the other hand, the very conditions under which Deposit Contributors were to receive their benefits held out certain inducements to those in better health than the average, and in consequence it has been found that certain employed persons have from choice become Deposit Contributors. The advantage here alluded to is the return to the Deposit Contributor of four-sevenths (one-half in the case of women) of the amount standing to his individual credit on proving to the satisfaction of the Insurance Comissioners that he has permanently left the United Kingdom. To a modified extent the promise of a similar proportionate payment in case of death was undoubtedly an inducement to some to elect to become Deposit Contributors. Neither of these benefits is obtainable by a member of an Approved Society. But it is probable that the vast majority of those who are at present Deposit Contributors are such simply because they have neglected to take the necessary steps to become members of societies, while it is undoubtedly true that a number have become Deposit Contributors in order that it may not become known that they are insured persons.

One thing at least is clear—namely, that the original anticipation that the Deposit Contributor class would consist mainly of those who were uninsurable has not been realised.

Again, the actual number of Deposit Contributors is considerably less than half the number originally estimated, the latest figures showing that there are about 360,000, whereas over 880,000 was the expectation as shown in the actuarial estimates.

The foregoing facts indicate that the problem of how to deal with the Deposit Contributor, when it comes to be reviewed by Parliament, will be very different from that originally anticipated.

### Specific Instances.

So far we have dealt with the matter quite generally, but instances can be given which throw into relief some of the difficulties of arriving at a satisfactory solution. For example, Mr. Kingsley Wood, a member of the London Insurance Committee, is reported to have called attention, in a paper read before the Faculty of Insurance on March 7, to the fact that the percentage of Deposit Contributors to total insured persons is twice as large in Hampstead as it is in Bethnal Green, while the largest percentages came from the City of London, Holborn, Paddington, and Westminster. This shows conclusively that as a class the Deposit Contributor is not so wholly devoid of the power of looking after their own interests as might have been imagined. If further evidence were required of the fact that as a whole the class is above the normal of the Approved Societies in the standard of health, we could not do better than refer to the paper recently published in the *National Insurance Gazette*, wherein the Clerk to the Hull Insurance Committee reports that in his area, during the past year, the sickness claims among Deposit Contributors were exceedingly light.

On the other hand, it is admitted that the system of individual accounts, as opposed to a common insurance fund, is of very little use in the case of protracted illness, and as a provision for maternity benefit it is absolutely inadequate. "In London alone," quoting again on the authority of Mr. Kingsley Wood as reported in the *Times*, "over 15 per cent. were disqualified from benefit by January 14 last owing to their accounts being exhausted." Many more would doubtless fall into the category of those who would only be entitled to receive reduced benefits in the event of sickness or upon the birth of a child.

### Difficulties in the Way of a Solution.

It was the intention of the framers of the Act that the present arrangements should be reviewed by Parliament with a view, if possible, of removing the disabilities under which Deposit Contributors are placed when they have drawn benefits and thereby exhausted their credit. And, although the class is very differently constituted on the whole from that originally contemplated, indications are not wanting to show that a sufficiently strong case can be made for the revision of the Act. It would, of course, be possible for the existing plan to be continued, perhaps for an indefinite period, by means of the Expiring Laws Continuation Act, but for the reason already stated this method of procedure is not likely to be adopted.

What, then, is to be the solution?

In order that insurance may be effective there must be some form of combination, and this is just where the difficulties are likely to arise. If no special society be formed to take over the whole of the Deposit Contributors, then some form of compulsion would have to be exercised—the contributors would be compelled to find societies, and the societies would be forced to accept them. To such a course there are obvious disadvantages on both sides, besides the difficulties that would be involved in adjusting the terms upon which individual transfers are to be made, when some have funds to their credit, while others have long since exhausted their rights to benefit.

If, on the other hand, it be determined to bring into existence a Special State Society, managed by the Insurance Commissioners, or by other Government officials, it is probable that considerable opposition would be raised by existing Approved Societies.

A State Society would necessarily imply a State guarantee, and this is not given to existing societies.

Furthermore, although it has been stated on high authority that the Friendly Societies are desirous of being relieved of the administration of National Health Insurance, they are by no means anxious to quit the field unless all classes of Approved Societies are absorbed by State management, and they would stand with the Approved Societies generally against any partial scheme of State management. Such a change as would be implied by the transference of the Approved Societies to direct Governmental control would not only be a radical alteration of the fundamental basis of the present system, but it would also appear to involve additional State aid, and for this reason it is not likely that any general alteration in the control of the societies will be made. Sufficient has been said to indicate the difficulties that now confront the Government in devising a statesmanlike plan for dealing with the Deposit Contributor class—a plan which should no longer be merely a temporary expedient, but a satisfactory permanent scheme, designed with due regard to the well-being of the community as a whole, as well as that of the persons more immediately affected.

